# The origin of cost–benefit analysis: a comparative view of France and the United States

**DOI:** 10.1186/s12962-021-00330-3

**Published:** 2021-11-18

**Authors:** Wei Jiang, Rainer Marggraf

**Affiliations:** 1 State Key Laboratory of Urban and Regional Ecology, Research Center for Eco-Environmental Sciences, Chinese Academy of Sciences, No. 18 Shuangqing Road, Haidian District, Beijing, 100085 China; 2 Department of Agricultural Economics and Rural Development, University of Göttingen, Platz Der Göttinger Sieben 5, 37073 Göttingen, Germany

**Keywords:** History, Early development, Cost–benefit analysis, France, United States

## Abstract

**Background:**

Cost–benefit analysis (CBA), as a common instrument in the decision making process on how to allocate financial resources, has been widely used in various research areas and in almost all of countries over the world. However, the origin and the historical development of CBA has long been subject to neglect. We attempt to fill this gap and clarify the origin and the early development of CBA.

**Methods:**

A comparative analysis is used to investigate the origin and the early development of CBA in France and the USA. The comparison is focused on two questions: (1) which criteria should be applied to decide whether or not a project should be carried out, and (2) with which procedure these criteria can be used for real projects.

**Results:**

The origin of CBA can be dated back to the work of Saint-Pierre in France in 1708. Dupuit introduces the concept of consumer’s surplus that founds the economic basis of CBA. These works are not taken seriously in France and do not draw attention from other countries. Hence, until the 1930s, the principle of CBA is newly proposed in the US and the Green Book marks the mature of CBA.

**Conclusions:**

The early development of CBA in France and the US is independent from the aspects of historical background, personnel, approaches and standardization. This study could help researchers of various disciplines be sure about the history of CBA when they perform this analysis in their research areas.

## Introduction

Cost–benefit analysis (CBA) is defined as a systematic cataloguing of impacts as benefits (pros) and costs (cons), valuing in dollars with assigned weights, and then determining the proposal relative to the status quo by the net benefits (benefits minus costs) or the benefit–cost ratio (divide benefits by costs) [4]. CBA is a decision-aiding tool that quantifies in monetary terms the value of all consequences associated with a government policy (such as setting an environmental standard) or with an investment project (such as reforestation in a floodplain) to all members of society. The purpose of CBA is to help social decision making and to allocate scarce resources more efficiently [23].

**Fig. 1:**
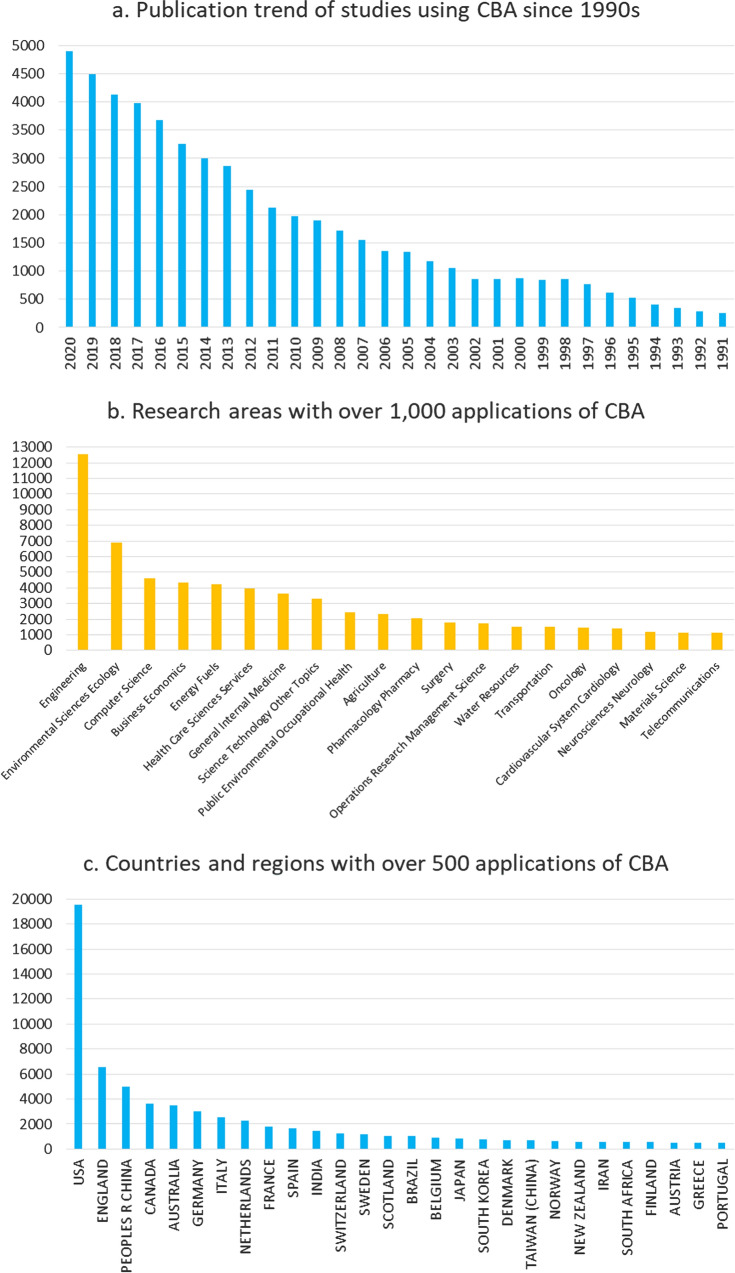
**a** Publication trend of studies using CBA since 1990s; **b** Research areas with over 1000 applications of CBA; **c** Countries and regions with over 500 applications of CBA.

By 2020, CBA has been widely applied to various research areas in almost all countries over the world. Based on the Web of Science Core Collection, the search of cost–benefit analysis in the topic during the period 1900–2020 (performed on September 23, 2021) results in a total of 54,445 publications. The first application of CBA was published in 1951, and studies using CBA have seen significant increase since the 1990s (Fig. 1a). CBA has been used in 146 research areas, among which the most applications with over 12,000 were found in engineering, followed by environmental sciences ecology, computer science, business economics, energy fuels, health care sciences services, and internal medicine (Fig. 1b). CBA has been used in 197 countries and regions, among which the most applications with nearly 20,000 were found in the USA, followed by England, China, Canada, Australia, Germany, Italy, Netherlands, and France (Fig. 1c).

Unfortunately, the origin and the historical development of such a widely used approach has long been subject to overt neglect. There are three opinions about its origin in the literature. Most of researchers take the position that CBA first emerged in the United States during the 1930s (e.g., [22, 28]). Hanley and Spash [24] were the first, and Boland et al. [5] followed, to claim the earlier origin by Albert Gallatin of the US Secretary of the Treasury in 1808. And, Pearce [40] and Pearce et al. [41] attributed its origin to the work of Jules Dupuit in 1844. This makes confusions but indicates that CBA possibly originated in France or the USA.

Therefore, in this paper, we attempt to fill this gap and clarify this confusion by tracing out the origin of CBA and the early development of calculation methods in France and the US, respectively. This study could help researchers of various disciplines be sure about the history of CBA when they perform this analysis in their research areas.

## Methods

The comparative analysis method is used to investigate the origin of CBA. The comparison is focused on two questions: (1) which criteria should be applied to decide whether or not a project should be carried out, and (2) with which procedure these criteria can be used for real projects. Ekelund and Hébert [16] and Hufschmidt [28] offered insightful information on the considerations of both questions in France in the eighteenth and nineteenth centuries, and in the United States at the beginning of twentieth century, respectively. Based on these two studies, we use the snowball strategy [29] to find more relevant literature, which are then comparatively analysed. In Section “The origin and development in France”, we elaborate the thinking and practices of French engineers, while in Section “The beginning and rise in America”, we investigate the procedures developed by American professionals. Finally, we conclude with the findings of the comparison in Section “Discussion and conclusion”.

## The origin and development in France

The idea of a national transportation network in France began in the seventeenth century, since when French engineers worked consciously at introducing a decision rule to guide the construction of public works. The earliest demands depended on French engineers by military considerations, the most prominent engineer of this era was Sébastien Vauban, whose key role was associated to his formative influence on the Corpes des Ingénieurs des Ponts et Chaussées, which was instituted in 1716. A central office within the Corps was established in 1747 for the purpose of training more men and improving their effectiveness, it gradually evolved into the École des Ponts et Chaussées in 1775 and was renamed the École Nationale des Ponts et Chaussées (ENPC) after the Revolution. At the ENPC mathematics was highly appreciated, courses were also given in the engineering subjects of road, bridge and canal building, flood control, harbour improvement, and railroad construction, and economic studies were incorporated into coursework early on. In the first half of the nineteenth century the graduates of the ENPC have been a well-trained group, referred to as Ponts engineers, who developed some fundamental principles and analytical tools to solve cost–benefit calculations of the construction of public works. Jules Dupuit was the culmination among those brilliant engineers in this field [16, 42].

### The pre-revolutionary period

The first formal cost–benefit study was undertaken in 1708 by the Abbé de Saint-Pierre in measuring the incremental benefits of road improvements. He theorized that incremental benefits would result from increased trade and reduced transport costs. The benefit of increased trade was calculated in two steps. First, the annual value of agricultural output in each province from the annual tax revenue collected was estimated. Then a loss factor, percentage of annual output not produced due to the impossibility of transport, was added to this magnitude. The resulting figure indicated the benefit of increased trade because this loss would be restored by improving the roads. The benefit of reduced transport costs was calculated by the savings per horse and driver. Assuming that the roads were equally passable in all seasons, horses would be able to carry 20 percent more weight, and more trips could be made, only 80 percent as many horses would be required as before the road improvements, resulting in annual savings of 20 percent fewer horses and drivers. On the cost side, the annual additional expenses consisted of costs for administration, repair and continuing maintenance [16].

From the very beginning of CBA, Saint-Pierre was sensitive to the use of incremental analysis in evaluating public goods. He was also alert to the indirect of secondary benefits by observing that better roads could attract industry and trade, which in turn could increase employment. Additionally, he was well aware that this kind of analytical techniques could be fruitfully applied to all public works. Saint-Pierre framed the important issues that were confronted by generations of Ponts engineers, and his argument was accepted by the engineers of the newly formed Corps [16].

The era of canal construction in pre-revolutionary France gave the engineers the possibility to turn their cost–benefit calculations to greater effect because the benefits and costs of canals are more precisely economic and more susceptible to measurement. However, due to technical and financial difficulties, the wave of canal construction failed to stimulate much analytical progress. A project for constructing a canal must be certified by engineers according to the public utility of the canal, but the engineers at that time did not understand the concept of demand. Benefits could neither be calculated by increased trade nor by reductions in transport costs, because no trade had existed before. Therefore, benefits were identified chiefly in terms of value of time saved in the shipment of goods, which was notoriously difficult to deal with. Two decision rules emerged eventually to solve these difficulties. First, a canal produced net utility when the resulting savings in transport costs were greater than its construction costs, and second, a canal adds utility when, treating construction costs as sunk, its toll revenues exceed its maintenance costs. But the appropriate level of tolls posed another vexing problem because any levy reduced the public utility of canals [16].

### The revolutionary period

The revolutionary spirit made all past institutions suspect, including the Corps and the École, so that the old regulations in relate to public works were in part abolished, in part abandoned, and altogether dependent on local conditions. After ascending to power Napoleon Bonaparte helped restore the order to the administration of the Corps and began to assign many new projects, most of which were driven by political or military considerations. He was mainly concerned with cost and speed of project construction, therefore, the engineers’ attention was focused on cheapness and expediency rather than on calculating expected benefits. The problem of minimizing transport cost had been solved mathematically by Gaspard Monge in 1776, but this was not a complete solution to the problem at hand because it shed no light on the issue of benefits. It is just on the benefit side that real progress should be made [16].

### The post-revolutionary period

The engineers were recentralized in the Ministry of the Interior during the Restoration, making a fertile period in economic analysis from the 1820s to 1840s. The stimulus given to canal construction prompted a number of minor advances in the formulation of CBA.

In 1822, focusing on the value of time saved in transport and the amortized costs of building and maintaining a canal, Pierre-Simon Girard tried to measure the benefit of the canal in physical terms by employing a curious combination of hydraulics and economics. The shortcomings of his method were demonstrated by Louis-Joseph Favier in 1824, who established the principle that a public work could be justified when it conveyed positive net utility, that is, the amount of net revenue from the public work must be greater than the cost of (re)construction, disregarding how the revenues and costs were assigned. To emphasize the choice between alternative public investments Favier derived a rule stating that a public work is to be preferred if its net utility exceeds that of another, taking into account the life of the respective constructions and amortized costs [16].

As early as 1830, a prominent Ponts engineer named Henri Navier set up a cost–benefit principle that public works should be provided only if the total benefits exceeds the total costs by measuring the benefits of new transport facilities based on an estimate of cost savings. His effort produced a decision rule that allowed for calculating minimum demand for new public works, below which the construction would be against the interests of the state. Algebraically, Navier defined the annual recurrent costs related to a new canal (*C*), the price of goods transported by road (*r*), the price of goods transported by canal (*c*), cost savings to consumers attributable to the canal (*S* = *r* *−* *c*), and the annual amount of goods transported on the new canal (*n*). Since n is a function of S, there must be some *n*′ and *S*′ such that *n*′*S*′ = *C*, so n′ is the minimum demand being sought. If *n* > *n*′, the state would gain annually a net amount equal to *S* times (*n* *−* *n*′), while if *n* < *n*′, the state would lose annually a similar amount [13, 15, 16].

Joseph Minard, who was an important link between Navier and Dupuit in the development of CBA, made two major advances to Navier’s analytical framework in 1832. First, he recognized that utility-increasing cost savings resulted from changes in consumption by inducing old consumers to substitute the lower-priced good for other goods and by drawing into the market new consumers who could not afford the good before. When new consumers entered the market, the utility gained by society would depend on consumers’ reaction to this price change, which in turn would depend on the consumers’ income. Second, in comparison to Navier, Minard introduced more subjective elements into the measure of benefit. A unique one was his explicit treatment of time. He insisted that time must be given a value, and that failure to take the benefits from time saved into account would lead to systematic underestimate of social benefit. However, he was clearly aware of the difficulty in evaluating the time, and he overcame this hurdle by assigning a subjective monetary value to the time, for example using wages as the opportunity cost of a worker’s time [13, 15, 16].

In 1833, Charlemagne Courtois developed a single principle for the selection of the most preferable transport project linking two cities. That was to choose the project that, given the costs, provided the greatest benefit. To identify the benefits of a project, Courtois considered as relevant variables the amount of goods in tons (*n*), the transport costs per ton and kilometer (*p*), the distance of the route (*l*), a sum of outlay (*A*), and the construction and maintenance costs (*C*). His analysis distinguished between communications already existed and new ones to be established. In the first case, he argued that the most preferable project should be the one over which, for a given outlay *A*, the greatest amount of goods could be carried. Since *A* = *nlp*, he took n = *A*/*lp* as the typical form of the solution and concluded that to the least product of l and p would correspond the greatest value of n, and consequently the most preferable project. In the second case, the construction and maintenance costs should be taken into account. Courtois introduced the ratio of the amount of goods carried per unit of construction and the maintenance costs as the benefit criterion which he called “the measure of advantages”. He took *n*/*C* = *A*/*Clp* as the typical form to determine the character of the project with the maximum of advantages [16, 46].

André Mondot de Lagorce adopted and improved these considerations by more rigor and generality in 1840. He treated costs more sophisticatedly by normalizing annual maintenance costs in terms of the average costs of labor and materials, and he was keenly aware of the difference between the interest rate as a cost of capital and the discount rate used to reduce future expenditures to present value. Mondot realized that a full solution required estimating transport demand on the new route, and admitted that it was impossible to determine the exact demand a priori, because demand depended on the choice of projects, which was the solution being sought. But he refused to abandon economic calculation, insisting that the estimation of demand, despite imperfect, was better than arbitrary decision [16].

On the cost side, Mondot defined the construction costs (*c*), the annual maintenance costs (*d*) normalized according to average costs of labor and materials, the appropriate discount rate (*r*), and the annual savings in maintenance costs on the old road (*S*) owing to less traffic after the new road is built. Thus, the annual expense of the new road (*C*) is equal to *cr* + *d* *−* *S*. On the benefit side, he defined the average transport cost on the new road (*p*) calculated as a function of weight, the average transport cost on the old road (*q*), and the estimated amount of goods to be transported on the new road (*n*). Thus, the total benefit of the new road (*B*) is equal to *n*(*q* *−* *p*). For the value n was not given, Mondot proposed the measure of “the real utility per unit of expenditure”, which is equal to (*q* *−* *p*)/*C*, as “the administrative value” of a project. What he called a “normal project” was the one with the highest “administrative value”. Mondot’s work typified the simple definition of CBA by proposing a criterion that compared disadvantages (*C*) with advantages (*B*). So long as a large number of projects were desirable, rigorous estimate of transport demand (*n*) on new routes was not always a pressing problem, for one could simply reject all projects for which *B* *−* *C* was not sufficiently positive [16].

However, this straightforward approach ignored important demand effects that resulted from the reduction of commodity prices induced by lower transport costs. A more sophisticated solution required a theory of demand. In 1844, Jules Dupuit published his breakthrough article “On the measurement of the utility of public works”, not only providing the demand function derived from a basic theoretical principle of consumer behaviour, i.e., marginal utility, but also introducing a practical measure of economic welfare, i.e., consumer surplus, which became the theoretical basis of CBA and stood as lasting monuments to the pioneer efforts of the French engineer-economists [16, 45].

By raising the question of how the utility of public works was to be measured, Dupuit began with the definitions of utility. Using the examples of wine tax (market good) and water system in a town (public works), he came to the conclusion that “each consumer himself attaches a different utility to the same thing according to the quantity which he can consume”. Then he distinguished between the absolute utility and the relative utility. “In general, the relative utility of a product is expressed by the difference between the sacrifice which the purchaser would be willing to make in order to get it, and the purchase price he has to pay in exchange”, supposing that the market price of the product is more or less equivalent to the costs of production. Although Dupuit did not identify the exact concept of marginal utility, he did illustrate the idea from an empirical consideration that Ponts engineers typically confronted, and concluded that “in general every rise of fall in price decreases or increases utility by an amount equal to this variation for those who are consumers in both situations; for those who disappear or who appear, the utility lost or acquired is equal to the old or to the new relative utility yielded to them by the product” [11, 14, 36].

**Fig. 2:**
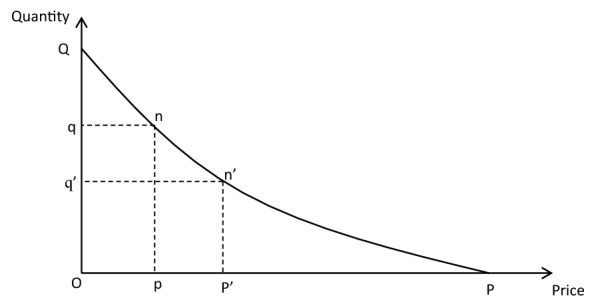
Dupuit’s “consumption curve”

By pointing out the error in Navier’s calculation of the utility of a canal, Dupuit proposed his method, arguing that the measure of utility for products already being consumed should be based on reduction in costs of production rather than reduction in costs of transportation, while in the case of new commodities being transported the measure of utility would be the lowest tax which would prevent their being carried by the new route. To operationally calculate the utility of public works, Dupuit derived his “consumption curve”, which was actually the marginal utility curve (Fig. 2). He defined the “consumption curve” as *q* = *f*(*p*), thus placing the independent variable (price) on the x axis and the dependent variable (quantity) on the y axis. He showed that the absolute utility of *Oq*′ articles is equal to the area *Oq*′*n*′*P* under the consumption curve, and derived the relative utility, what is now called consumer surplus, by subtracting the costs of production shown as *Oq*′*n*′*p*′, which leaves the area *n*′*p*′*P*. Suppose the price decreases from *p*′ to *p* due to a reduction in costs of production, so that the quantity consumed increases from *q*′ to *q*. This raises the absolute utility to *OqnP*, subtracting costs of production *Oqnp* from this amount yields the relative utility of *npP*, so the net gain in relative utility is measured by *pnn*′*p*′ [11]. In this manner Dupuit not only developed a monetary measure of the benefit of public works and of goods in general, but also forged the most important tool of welfare economics. It was a significant breakthrough, but clearly far from perfect.

### Summary

We summarize the contributions to CBA made by French engineers in Table 1. Unfortunately, the methods for calculating benefits proposed and improved by the engineers did not form general standards or decision rules that were accepted on the administrative level to value a single project or to justify the choice between rival projects. Until the end of the nineteenth century the administrative form of economic quantification in project planning was still carried out in terms of cost and revenue, not costs and benefits [42]. However, on the other side of the Atlantic, CBA was emerging and would see its real rise in the United States of America.

**Table 1:** Contributions of engineers to the development of CBA in France

Year	Person	Contribution
1708	Abbé de Saint-Pierre	Theorizing that extra benefit of road improvements is equal to benefit from increased trade plus benefit from reduced transport cost minus additional expenses for improvement
1776	Gaspard Monge	Solving the problem of minimizing transport costs mathematically
1822	Pierre-Simon Girard	Measuring the benefit of a canal in terms of the value of time saved in transport and the amortized costs of building and maintaining the canal
1824	Louis-Joseph Favier	Establishing the principles that the amount of net revenue from a public work must be greater than the cost of (re)construction, and that a public work is to be preferred if its net utility exceeds that of another
1830	Henri Navier	Setting up the cost–benefit principle that public works should be provided only if the total benefits in terms of cost savings exceeds the total costs
1832	Joseph Minard	Making two advances to Navier's work by recognizing that savings resulted from changes in consumption and by introducing the value of time into the measure of benefit
1833	Charlemagne Courtois	Developing a single principle for the selection of the most preferable transport project linking two cities by calculating the greatest benefit given the costs
1840	André Mondot de Lagorce	Typifying the cost–benefit principle that compares advantages with disadvantages of a public work
1844	Jules Dupuit	Establishing the demand function based on marginal utility and introducing a practical measure of economic welfare known as consumer's surplus

## The beginning and rise in America

The American water resources development, including navigation, flood control, irrigation and water power, initiated from the beginning of the nineteenth century [20, 49]. The Gallatin Report of 1808 proposed partly a nationwide system of canal and river improvements justified on the basis of economic development of the west, political unity and national defense needs, but it had no immediate effect because it was only a statement about the issue of public investment in transportation and far from an economic analysis of individual projects [25, 27].

As the first major construction agency with the requisite technical abilities, the Army Corps of Engineers established officially in 1802 on the model of the Corpes des Ingénieurs des Ponts et Chaussées, was given the responsibility for planning river and harbour improvements in 1824. The establishment of the Mississippi River Commission involved the Corps in flood control in 1879. The 1902 River and Harbor Act created a national-level Board of Engineers within the Corps to evaluate construction and maintenance costs, commercial benefits and necessity of river and harbour improvements. The 1917 Flood Control Act introduced the principle of local financial contributions to flood control, and authorized the Corps to undertake comprehensive studies of watersheds regarding the relationship of flood control to navigation, water power and other uses. The 1920 River and Harbor Act further required the reporting of local benefits for the recommendations of appropriate local cost sharing [1, 27, 47]. American efforts to economic evaluations of public investments during this early era were lack of rigor and depended almost completely on estimate [25, 42]. The Report on the Chesapeake and Ohio Canal made in 1826 is a representative example (Table 2).

**Table 2:** The costs and benefits for constructing the Chesapeake and Ohio Canal () modified according to Board of Internal improvement [3]

Cost				
section	Distance (km)	Ascent or descent (m)	Number of locks	Estimated cost ($)
Eastern	298.7	176.2	74	8,177,081.05
Middle	113.6	597.7	246	10,028,122.86
Western	137.1	188.7	78	4,170,223.78
Sum	549.4	962.6	398	22,375,427.69

Benefit	Estimated benefit ($)
Augmentation in the value of lands, or benefit derived by owners of real property	36,780,000
Total of successive augmentations of the value of the products during 6 years, or advantages obtained by the producers	38,989,560
Total of successive augmentations by the revenue of the customs, during the same period	3,996,195
Benefits derived to commerce and the carrying business together, and for 6 years	1,859,830
Sum	81,625,585

### The beginning of CBA

The modern economic analysis of project value began during the New Deal. The most important agency in relation to water resources in this period were four successive national resource planning organizations operating between 1933 and 1943, namely National Planning Board (NPB, 1933–1934), National Resources Board (NRB, 1934–1935), National Resources Committee (NRC, 1935–1939), and National Resources Planning Board (NRPB, 1939–1943) [10]. With the most quoted passage “…if the benefits to whomsoever they may accrue are in excess of the estimated costs…” [48], the 1936 Flood Control Act is usually considered as the beginning of cost–benefit analysis in the United States [8, 22, 28, 40]. However, the outstanding report of the NRB in 1934 has the larger authority on the emergence of CBA for three reasons. First, like the famous act, this report had a clear statement that “we hope in general to achieve rational planning and in particular to achieve equitable allocations of benefits and contributions to cost in public works programs”. Second, it identified tangible, measurable intangible, as well as immeasurable benefits. Third, it included substantial economic basis. Additionally, two principal categories of water projects were recognized, one for income producing e.g., hydroelectric power and another for loss preventing e.g., flood control, which implied that prevented tangible and intangible losses are the measure of benefits [37]. Nevertheless, the 1936 Flood Control Act still has significant meaning that a strict cost–benefit rule is written into law and hereafter Congress can only, without exceptions, authorize projects that have been studied and approved [42].

The 1938 report of the NRC suggested that social and economic benefits, general and special benefits, potential and existing benefits should be taken into account in deciding whether or not large water projects should be undertaken as well as in distributing the costs of projects among the beneficiaries [38]. The 1941 report of the NRPB further recognized two general categories—tangible and intangible—of benefits and costs as well as two types—primary and secondary—of tangible benefits and costs. It suggested that a project plan was economically sound if total benefits were greater than total costs, and benefits from each function of multiple-purpose projects were greater than separable costs incurred solely in serving that function [39].

The Water Resources Committee of the NRPB and its predecessors contributed greatly to the development of CBA. Although much of its contribution was not highly technical and far less than complete [10], the committee’s works were so basic and influential that they would consistently find retrospective in the postwar history of CBA. However, CBA itself was only an administrative device owing nothing to economic theory in this initial phase [21] and did not become the principal basis for project evaluations of related agencies until the 1950s.

### The issuing of the Green Book

Besides the Corps, there were many other agencies involved in water resources development, however, each agency adopted different and inconsistent methods of estimating costs and benefits. The 1941 report drew attention to these inconsistencies and advocated cooperative studies to develop uniform methods. After the NRPB was abolished in 1943, a new pattern of coordination arose with the establishment of the Federal Interagency River Basin Committee (FIARBC). In 1946 a subcommittee on benefits and costs was appointed “for the purpose of formulating mutually acceptable principles and procedures for determining benefits and costs for water resources projects”. This subcommittee issued a final report entitled “Proposed Practices for Economic Analysis of River Basin Projects” in 1950, which became known as the Green Book [27, 42].

The Green Book is recognized as the first landmark in the history of CBA in the United States [27, 28]. CBA covered completely for the first time its modern subjects, including definition of benefits and costs, general procedure for the measurement, consideration of available alternatives, criteria for comparing alternatives, choice of discount rate, risk allowances, and economic life of projects. One of the strengths of the Green Book lies in stating the basic principles of microeconomics, although not in highly theoretic terms. It stated that the ultimate aim of water resources development projects is to satisfy human needs and wants by providing goods and services, which refer to all objects and activities that have the power of satisfying human needs and may increase or decrease in availability as a result of a project. It was aware of the limitations of the market price system in reflecting values of goods and services from a public viewpoint, but concluded that there is no more suitable framework for evaluating public projects in common terms. Therefore, market prices had to be chosen as the starting point for measuring the tangible effects of a project, whether benefits or costs. Some tangible effects that cannot be assessed based on market prices may be derived indirectly from prices for analogous effects or from the most economical costs of producing similar effects by an alternative means [18].

Another advantage of the Green Book is to apply these principles to develop operational procedures for quantifying benefits of various project purposes, such as irrigation, flood control, navigation, electric power, and recreation, although not in sufficient detail to serve as a manual. For example, the primary benefits of flood control should either be measured in terms of the estimated costs that would be avoided with flood control but would be incurred without it, or be evaluated as the costs of repairing or rehabilitating the affected property. Measuring primary benefits from water power was based on the costs of equivalent power from alternative sources that would most likely be utilized in the absence of the water power [18].

Here, all of cost-based methods that are used widely today, namely avoided cost method, restoration cost method, and replacement cost method, have their rudiments. Unfortunately, the Green Book argued that the benefits of navigation improvements were measured by savings in transportation costs rather than reduction of production costs, which was Dupuit’s main point before one hundred years. For the purpose of evaluating recreational benefits, the Green Book mentioned two approaches being used at that time [18]: recreational benefits were assumed either to be equal to the sum of expenditures by recreationists for items like gasoline, lodging and equipment (expenditure approach), or to be equal to the costs of installing, operating and maintaining specific recreational facilities plus an equal amount that was considered to be the value of the benefits attributable to recreational use of facilities provided for purposes other than recreation, suggesting that recreation benefits were equal to twice the specific costs (twice-cost approach).

### The bureau of reclamation and the bureau of the budget

Although the Green Book had considerable influence, it failed utterly to reconcile the cost–benefit practices of relevant agencies, especially the Bureau of Reclamation (BOR). The BOR, established by the Reclamation Act in 1902 for the purposes of making examinations and constructing irrigation works, was the most important rival agency against the Corps in this field. Since the BOR was the specialist on irrigation, it created a set of discrepant methods for quantifying the benefits of irrigation, which contained extravagant measures of indirect benefits. Possibly because the BOR was not involved in preparing the Green Book, the report took gentle but clear position against the Bureau on the issue of secondary benefits, stating that secondary benefits should only be considered under certain strict conditions. It was no surprise that the BOR did not accept these restrictions [28, 42]. In order to resolve this issue, the BOR called on a Panel of Consultants to indicate the adequacy of existing procedures for evaluating secondary benefits, and to set forth a recommended basis for their evaluation. The report of the Panel in 1952 stated that secondary benefits were much less determined and measurable than primary, and depended more on far-reaching hypotheses. Usable formulas could not be based on data that are capable of affording accurate and complete comparisons of effects with and without a given project. Thus, it recommended that primary and secondary benefits be separately shown in benefit–cost ratios [9].

Beginning in 1943, the Bureau of the Budget (BOB) was required to review and consolidate all public works including water resources projects. Attempting to supervise the economic justification of projects, the BOB in 1952 issued Budget Circular A-47, which was in many respects similar to but more restrictive than the Green book, to set forth uniform standards and procedures in reviewing proposed water resources projects [27, 42]. The Budget Circular A-47 based the evaluation mainly on primary benefits, and provided that not only must the total benefits of a project exceed its costs, but the benefits attributable to any purpose of a multi-purpose project must exceed the costs of including that particular purpose. Additionally, it proposed clearly for the first time that increases in the values of recreation and fish and wildlife resources as a result of the project were a category of primary benefits to be included in evaluation [7].

### Summary

The early work on water resources evaluation until the early 1950s was undertaken by professionals from federal agencies (Table 3). Many of these professionals had had a bureaucratic as well as training identity that they worked in the Bureau of Agricultural Economics. However, academic economists relevant to CBA outside the bureaucracy did not almost exist in the early 1950s. There was very few papers published in economic journals on the economics of public investments, and the work of these agricultural economists on the benefits of public works was more closely related to a bureaucratic discourse than an academic one [2, 28, 42].

**Table 3:** Contributions of federal guidelines to CBA in America

Year	Document	Contribution
1826	Report on the Chesapeake and Ohio Canal	Providing the typical analysis approach based on estimate for public investment
1934	Report of National Planning Board	Marking the beginning of cost–benefit analysis in America
1936	Flood Control Act	Writing the strict cost–benefit rule into law
1950	Proposed practices for economic analysis of river basin projects (the Green Book)	Covering all subjects of modern CBA, establishing all cost-based methods for measuring benefits
1952	Report of Panel of Consultants to the Bureau of Reclamation	Discussing the issue of secondary benefits
1952	Budget Circular A-47	Including recreation as a category of primary benefits

Along with the consolidation of welfare economics in the 1950s [19, 32], CBA became quickly an attractive area for academic economists since the late 1950s. Two important institutions during this period, the Harvard Water Program and the RAND Corporation in California, generated an extensive literature of systematic studies [12, 26, 31, 33, 34]. Taken together, these books set a sound microeconomic and related welfare economic foundation for the theoretical and applied aspects of CBA. The various market-based valuation methods introduced in the Green Book were firmly established. In addition, some difficult conceptual issues such as externalities, opportunity costs, consumer surplus, and secondary benefits that were not familiar to or had troubled earlier practitioners were discussed and clarified [22, 28].

In the 1960s, the applications of CBA had widened from water resource projects to almost all kinds of government activity, such as public health [50], transportation [35], education [6] and urban renewal [43]. By this time, CBA had become not only a standard tool for the analysis of government expenditures, but also a legitimate branch of welfare economics [22, 42]. A still more significant breakthrough made by economists lies in the attempt to value recreational benefits, which leads to the flourishing developments of various environmental valuation techniques.

## Discussion and conclusion

The origin of CBA can be dated back to the work of Saint-Pierre in 1708. Through the efforts by a number of French engineers, Dupuit introduces the concept of consumer’s surplus in 1844 that founds the economic basis of CBA and measures benefits in terms of the reduction of production costs. However, these works are not taken seriously in France, and do not draw attention from other countries. Hence, the principle of CBA is newly proposed by the 1936 Flood Control Act in the US, and the Green Book in 1950 marks the mature of CBA by establishing cost-based methods for measuring benefits.

The comparative analysis shows that the early development of CBA in France and the US is independent from four aspects. First of all, there is no considerable evidence suggesting that the American experts are familiar with the early works of French engineers. Although Charles Ellet Jr., an American civil engineer, indeed traveled to Paris in 1830 to study as an external student at the École des Ponts, his contributions to the economic thought lie mainly in the practical problem of monopoly profit maximization of a railroad rather than CBA [16, 42]. The cost–benefit criterion proposed by Navier and Mondot finds no mention in the American documents. And the Green Book published in 1950 still considered the benefits of navigation improvements as savings in transportation costs rather than reduction of production costs.

Second, the backgrounds for introducing CBA in France and America are clearly different. The French tradition rooted in the field of transport economics, whereas the American tradition was related to water resources projects. Third, the personnel who made efforts to the development of CBA are also distinct. French engineers have formal academic background, but American professionals work in federal agencies. Both facts result in that French engineers follow a theoretical approach and attached great importance to mathematical calculation, while American professionals adopt an empirical approach and pay more attention to practical application [30]. This divergence in approaches leads to the theoretical foundation of consumer’s surplus in France and the practical guidelines of CBA in the United States, respectively.

Finally, the most crucial difference lies in the attempt for standardizing this approach in America, but such a progress does not take place in France. The political rival situation among agencies with overlapping responsibility is the major driving force for the standardization in the US [42], but the French Corps has a strong administrative, institutional and legally acknowledged monopoly position, which prevented the standardization. Another reason could be the lack of incentive within the power-conscious elites, the active thinking exchange among French engineers through the internal journal generate a variety other than a unity of measurement suggestions. More important, different from the practical considerations of American agencies, for which a pure quantitative economic excess of benefits over costs is necessary and sufficient, French decision-makers at that time also consider unquantifiable variable such as security, reliability, or even promotion of administrative centralization [17, 44].

## Data Availability

All data generated or analysed during this study are included in this published article.
